# The impacts of diagnosis-intervention packet payment on the providers’ behavior of inpatient care—evidence from a national pilot city in China

**DOI:** 10.3389/fpubh.2023.1069131

**Published:** 2023-06-01

**Authors:** Yi Ding, Jia Yin, Chao Zheng, Simon Dixon, Qiang Sun

**Affiliations:** ^1^Center for Health Management and Policy Research, School of Public Health, Cheeloo College of Medicine, Shandong University, Jinan, China; ^2^NHC Key Lab of Health Economics and Policy Research, Shandong University, Jinan, China; ^3^Health Economics and Decision Science (HEDS), ScHARR, University of Sheffield Regent Court, Sheffield, United Kingdom; ^4^Wits Centre for Health Economics and Decision Science, University of the Witwatersrand, Johannesburg, South Africa

**Keywords:** case-based payment method, diagnosis-intervention packet (DIP) payment, inpatient care provision, interrupted time series analysis, China

## Abstract

**Background:**

In 2020, the Chinese government developed and implemented an innovative case-based payment method under the regional global budget called the diagnosis-intervention packet (DIP) payment to pay for inpatient care. This study aims to assess the changes to inpatient care provision in hospitals after the DIP payment reform was implemented.

**Methods:**

This study used inpatient medical costs per case, the proportion of the out-of-pocket (OOP) expenditure in inpatient medical costs, and the average length of stay (LOS) of inpatient care as outcome variables, and conducted an interrupted time series analysis to evaluate changes after the DIP payment reform. January 2021 was taken as the intervention point when a national pilot city of the DIP payment reform in the Shandong province began using the DIP payment to pay for inpatient care of secondary and tertiary hospitals. The data used in this study were obtained from the aggregated monthly claim data of inpatient care of secondary and tertiary hospitals.

**Results:**

Compared to the pre-intervention trend, the inpatient medical costs per case, the proportion of the OOP expenditure in inpatient medical costs both in tertiary and secondary hospitals significantly decreased after the intervention. After the intervention, the reduction in the inpatient medical costs per case, the proportion of the OOP expenditure in inpatient medical costs in tertiary hospital were both higher than those in secondary hospital (*p* < 0.001). The average LOS of inpatient care in secondary hospital significantly increased after the intervention, and it immediately increase 0.44 day after intervention (*p* = 0.211). Moreover, the change of average LOS of inpatient care in secondary hospital after intervention was opposite to that in tertiary hospital, it had no statistical difference (*p* = 0.269).

**Conclusion:**

In the short term, the DIP payment reform could not only effectively regulate provider behavior of inpatient care in hospitals, but also improves the rational allocation of the regional healthcare resources. However, the long-term effects of the DIP payment reform need to be investigated in the future.

## Introduction

The growth of health expenditure has become a critical issue for healthcare policymakers and governments in many countries. To remedy this, countries have sought to improve the efficiency of healthcare delivery and reduce the growth rate of health expenditure by implementing healthcare provider payment reforms (1). Among the global empirical evidence of provider payment methods reforms, case-based payment approaches, such as the diagnosis-related group (DRG) system, have been widely adopted to pay for inpatient care (2, 3). Moreover, previous research has highlighted that case-based payment methods not only promoted the transparency and quality of healthcare services, but also enabled policymakers to not accurately compare the healthcare service performance of different hospitals (4).

Over past decades, total health expenditure (THE) in China has rapidly increased (5). This is thought to be a result of the prolonged use of fee-for-service (FFS) payments for inpatient care (6, 7), which has been associated with over-treatment and induced demand (8, 9). In an effort to curb the increase of the health expenditure, many cities throughout China have tried to design and implement various case-based payment methods (10, 11). The design frameworks of these case-based payments were based on the characteristics of local healthcare service markets, and as a result, it is difficult to generate generalizable findings from their implementation. Consequently, the Chinese government initiated DRG payment system reform in 2019 to control the growth of health expenditure (12). When developing the reforms, it was noted that a number of studies showed that the DRG payment system cannot cover all forms of healthcare services, and it also leads to unintended provider behaviors, such as up-coding, cream skimming, or induced demand behavior (13, 14). In addition, it was recognized that the operation of the DRG payment system requires a high administrative cost and advanced information system (15). Accordingly, the Chinese government decided to develop an innovative case-based payment system with a uniform design framework, which was intended to avoid some of the problems associated with existing DRG payment systems (16). Eventually, in 2020, the Chinese government launched an innovative case-based payment method under the regional global budget called the diagnosis-intervention packet (DIP) payment.

However, as the DIP payment is a recent initiative. There are few studies about the effect evaluation of the DIP payment, which leads that the current studies have not been able to adequately demonstrate its impact on changes to inpatient care provision until now. Among the few related studies, Lai et al. (17) discovered that the inpatient medical costs per case and the medication costs of inpatient care were significantly decreased after the DIP payment was implemented in Guangzhou. However, Qian et al. (18) outlined that the implementation of DIP yet increases the inpatient medical costs per case. Accordingly, this study selects the pilot city of the DIP payment in Shandong province as case city and aims to explore the changes to inpatient care provision in secondary and tertiary hospitals after the DIP payment reform was implemented, which can comprehensively demonstrate the impacts of diagnosis-intervention packet payment on the providers’ behavior of inpatient care.

## The design framework of the DIP payment

As with the DRG payment system, the DIP payment includes a series of classifications of DIP disease groups. Based on the same major diagnosis principles and similar treatment procedures, the National Healthcare Security Administration firstly enacted the uniform score catalogue and classification standards of the DIP disease groups, containing 11,553 core disease groups and 2,499 comprehensive disease groups. Subsequently, according to the uniform classification standards of the DIP disease groups, the 71 pilot cities of the DIP payment reform throughout China were required to enact the catalog of core disease groups and comprehensive disease groups. Meanwhile, the pilot cities needed to formulate the average score of each local DIP disease group by using the average medical cost of all inpatient care cases in the last 3 years.

**Figure 1:**
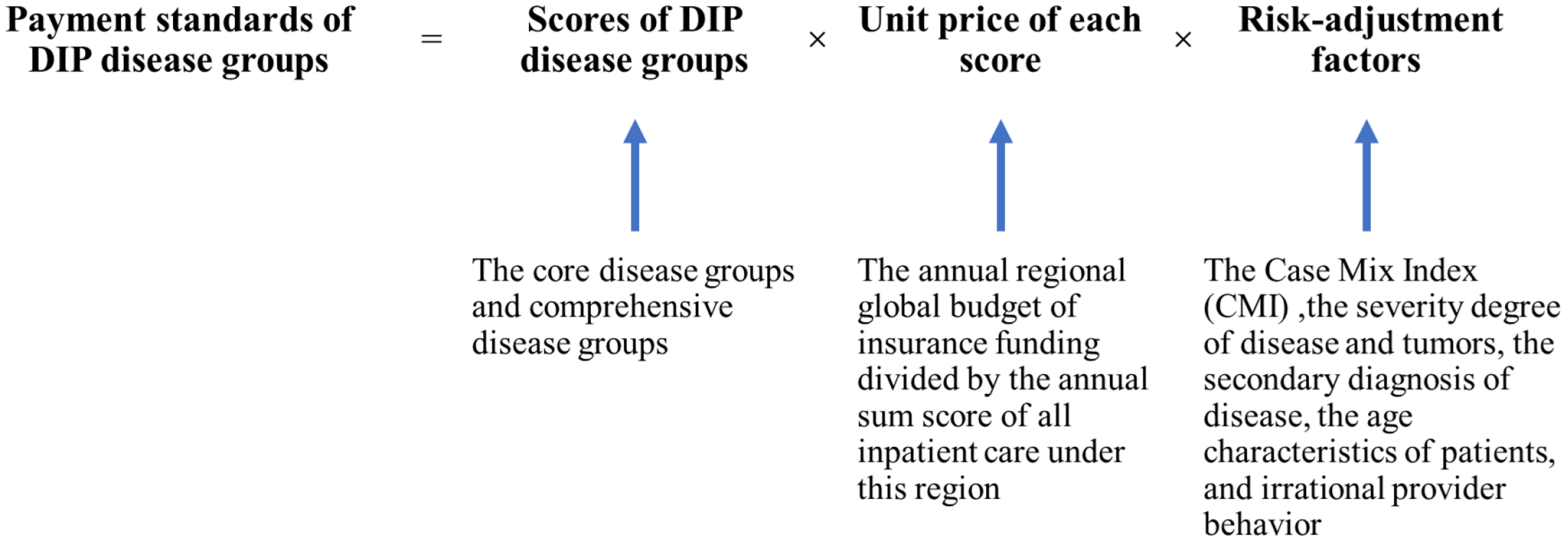
The basic design of payment standards of DIP disease groups.

Although the DIP payment has several differences with the DRG payment (Table 1), however the most significant feature of the new system is the design of payment standards of DIP disease groups (Figure 1). Unlike predetermined DRG payment systems, the payment standards of DIP payments are not fixed. Although the scores that reflect the consumption of healthcare resources of DIP disease groups are certain, the monetary value, or unit cost, of each score is not predefined. The value of the DIP payment depends on the regional global budget of insurance funding and the regional sum score of all inpatient care in the pilot cities (19). More specifically, the annual unit price of each score equals the annual regional global budget divided by the annual sum score of all inpatient care under this region, together with a further risk-adjustment (Equation 1). As such, if the healthcare service volumes increase, then the unit price of each score will be devalued, which will affect the revenue of the hospitals. The aforementioned risk-adjustment includes the Case Mix Index (CMI), the status of the hospital and the type of health insurance, are brought into the calculation of the reimbursements of hospitals.

**Table 1:** The differences between the DIP and DRG payment.

Differences	DIP	DRG
The number of disease groups	≥10,000	≤ 1,000
Grouping basis	Historical medical records	Clinical pathways (based on the experts’ experience)
Referring factors of formulating disease groups	Major diagnosis, related surgery	Patients’ gender, age, length of stay, clinical diagnosis, surgery, the severity of disease, complication and comorbidity, etc.
Variation between groups	Unobvious	Obvious
Payment unit	DIP groups	DRG groups
Payment standard	Unit price of each score	Relative weight of DRG groups

http://zzsybj.zaozhuang.gov.cn/ztzl/djqzpb/202204/t20220412_1415550.html.

Reimbursements to hospitals are further adjusted based on several indicators of performance. These include, quality of medical records, readmission rate, length of stay (LOS) and mortality. These adjustments are made in the form of organization level ‘fines’ (Equation 2).

(1) $$\begin{array}{l} \mathrm{Unit price of each score}=\mathrm{regional global budget of insurance funding}\\ \;\;\;\;\;\;\;\;\;\;\;\;\;\;\;\;\;\;\;\;\;\;\;\;\;\;\;\;\;\;\;\;\;\;\;\;\;\;\;\;\;\;\;\;\;\;\;\;\;\;\;\;\;\;\;\;\;\;\;\;\;\;\;/\mathrm{regional}\;\mathrm{sum}\;\mathrm{score of}\;\mathrm{all}\;\mathrm{inpatient care} \end{array}$$

(2) $$\begin{array}{l} \mathrm{Reimbursements of hospitals}=\mathrm{sum}\;\mathrm{score of}\;\mathrm{all}\;\mathrm{inpatient care}\\ \;\;\;\;\;\;\;\;\;\;\;\;\;\;\;\;\;\;\;\;\;\;\;\;\;\;\;\;\;\;\;\;\;\;\;\;\;\;\;\;\;\;\;\;\;\;\;\;\;\;\;\;\;\;\;\;\;\;\;\;\;\;\;\;\;\;\;\;\;\;\;\;\;\;\;\times \mathrm{unit price of each score}\\ \;\;\;\;\;\;\;\;\;\;\;\;\;\;\;\;\;\;\;\;\;\;\;\;\;\;\;\;\;\;\;\;\;\;\;\;\;\;\;\;\;\;\;\;\;\;\;\;\;\;\;\;\;\;\;\;\;\;\;\;\;\;\;\;\;\;\;\;\;\;\;\;\;\;\;\times rank\;\mathrm{coefficient}\\ \;\;\;\;\;\;\;\;\;\;\;\;\;\;\;\;\;\;\;\;\;\;\;\;\;\;\;\;\;\;\;\;\;\;\;\;\;\;\;\;\;\;\;\;\;\;\;\;\;\;\;\;\;\;\;\;\;\;\;\;\;\;\;\;\;\;\;\;\;\;\;\;\;\;\;\;\times \mathrm{risk}-\mathrm{adjustment factors}-\mathrm{fines} \end{array}$$

Through this design, the policymakers hope that the DIP payment can effectively incentivize individual hospitals to improve the efficiency and quality of their services, while promoting the allocative efficiency of regional healthcare resources.

## Methods

### Study design

As one of the 71 pilot cities in China, City A is the only pilot and typical city of the DIP payment reform in the Shandong province. It has three urban areas and two country areas. In 2021, the number of the resident population is 2.20 million residents, and the Gross Domestic Product *per capita* is 156.90 CNY which is the highest in Shandong province (20). Moreover, City A has seven tertiary hospitals and thirty-nine secondary hospitals that used the single case-based payment system under the regional global budget to pay for inpatient care before the DIP payment was introduced. The single case-based payment system in City A had many similarities to the DIP system that replaced it, however, important differences can be seen in relation to the number of disease groups, risk adjustment and payment ceilings for individual hospitals (Table 2).

**Table 2:** The design difference between the single case-based payment and the DIP payment in City A.

Elements of the payment	Single case-based payment	DIP payment
Payment scope	All local inpatient care cases	All local inpatient care cases of secondary and tertiary hospitals
Number of disease groups of payment	5,500 disease groups	3,210 DIP disease groups, comprising 2,765 core disease groups and 445 comprehensive disease groups
Principle of formulating disease groups	Major diagnosis	Same major diagnosis and similar treatment procedure
Principle of formulating scores of disease groups	Average medical cost of previous cases of all inpatient care in last 3 years	Average medical cost of previous cases of all inpatient care in last 3 years and experts’ suggestions
Calculation of the unit price of each score	Annual regional global budget/regional sum score of inpatient care	Annual regional global budget/regional sum score of inpatient care with a risk-adjustment mechanism
Annual ceiling of scores of hospitals	Setting the annual scores ceiling to different hospitals	Setting no annual scores ceiling to hospitals

In 2020, City A introduced the DIP payment system to categorize inpatient care without changing the reimbursement policies of inpatient care. From January 2021, City A started to use the DIP payment to pay for all inpatient care of local secondary and tertiary hospitals.

As such, the implementation of the DIP payment reform in City A was conceptualized as a quasi-experiment, with January 2021 serving as the intervention point in this study. An interrupted time series (ITS) design was used to assess whether the DIP payment reform produced significant changes to inpatient care provision in secondary and tertiary hospitals.

### Data sources

Fortunately, City A is the only one in Shandong province that did not have a COVID-19 pandemic before May 2022. All the residents’ healthcare consumption always can be satisfied in local hospitals. Therefore, we collected and aggregated the monthly medical claim data of inpatient care for secondary and tertiary hospitals in City A during January 2019 to April 2022, which was obtained from the Healthcare Security Administration in City A.

### Variables

To accurately quantify the change to inpatient care provision in hospitals, several variables were selected for measurement during the study observation, including inpatient medical costs per case, proportion of the out-of-pocket (OOP) expenditure in inpatient medical costs, and average LOS of inpatient care. All data in the study was adjusted using the related consumer price index, with 2019 serving as the base year (20). A log transformation was performed for the skewed data distribution in this study. Additionally, the ordinary least squares (OLS) regressions incorporated seasonal adjustments for the variables that displayed conspicuous seasonal effects (21).

### Statistical analysis

The ITS analysis was used to assess the changes to inpatient care provision. In the ITS analysis, a segmented OLS regression model with a Newey-West test was used to assess the impacts of the DIP payment reform on the change to inpatient care provision in hospitals (22). The segmented regression model we adopted is shown below:


$$Y_{t}=\beta_{0}+\beta_{1}T_{t}+\beta_{2}X_{t}+\beta_{3}T_{t}X_{t}+\varepsilon_{t}$$

*Y_t_* is the dependent variable we measured at every monthly point *t*, *T_t_* is the time series variable representing the time in months since the start of observation until to the time *t*, *X_t_* is a dummy variable representing the intervention point (pre-intervention period is 0 and the post-intervention period is 1), *T_t_ X_t_* is an interaction term of the time and intervention, and *ε_t_* is the residual term representing the unknown variation component of the regression model. *β_0_* represents the baseline level; *β_1_* represents the baseline trend prior to intervention; *β_2_* represents the immediate level of change after the intervention compared to the pre-intervention; *β_3_* represents the trend change after the intervention compared to the pre-intervention; and *β*_1_+ *β_3_* represents the trend after the intervention (23). The *actest* command was conducted to assess autocorrelation and the autocorrelation results were both present at lag1 (24). STATA 16.0 was used to perform all statistical analysis. Two-sided hypothesis tests of no effect, with *p* < 0.05 were considered statistically significant.

## Results

### Changes to the inpatient medical costs per case

**Figure 2:**
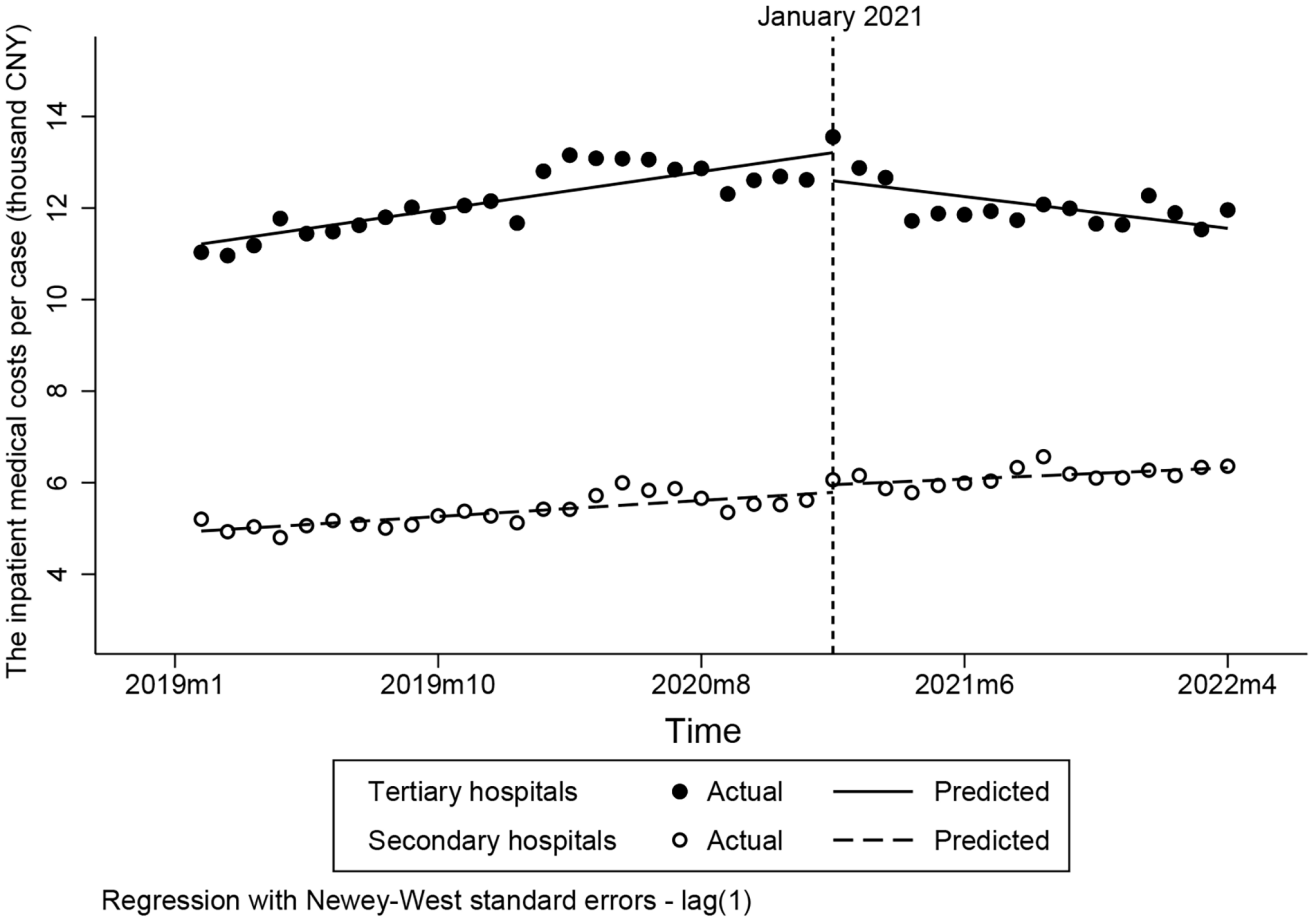
Changes to inpatient medical costs per case.

Figure 2 demonstrates that the inpatient medical costs per case in tertiary hospitals perceptibly decreased after the intervention. The inpatient medical costs per case in tertiary hospital decreased by 150 CNY per month compared to the pre-intervention trend (*p* < 0.001) (Table 3). Moreover, compared to the pre-intervention trend, the inpatient medical costs per case in secondary hospital decreased only slightly after the intervention, having no statistically significant difference (*p* = 0.307). However, the inpatient medical costs per case in tertiary hospitals keep decreasing by 0.07 CNY per month, which is opposite to that in secondary hospitals (*p* < 0.05) (Table 3).

**Table 3:** The change of levels and trends of variables before and after intervention.

Variables	Tertiary hospital				Secondary hospital	
	Coefficient	*P*	95% CI	Coefficient	*P*	95% CI
The inpatient medical costs per case (thousand CNY)						
*β_0_*	11.21	0.000	[10.97, 11.46]	4.94	0.000	[4.81, 5.08]
*β_1_*	0.08	0.000	[0.06, 0.11]	0.04	0.000	[0.02, 0.05]
*β_2_*	−0.61	0.167	[−1.50, 0.27]	0.17	0.287	[−0.15, 0.48]
*β_3_*	−0.15	0.000	[−0.22, −0.09]	−0.01	0.307	[−0.03, 0.01]
*β_1_ + β_3_*	−0.07	0.038	[−0.13, 0.00]	0.02	0.003	[0.01, 0.04]
The proportion of the OOP expenditure in inpatient medical costs (%)						
*β_0_*	45.02	0.000	[44.50, 45.53]	38.88	0.000	[38.57, 39.19]
*β_1_*	0.02	0.354	[−0.02, 0.07]	−0.06	0.001	[−0.09, −0.03]
*β_2_*	0.61	0.183	[−0.30, 1.53]	−0.32	0.419	[−1.13, 0.48]
*β_3_*	−0.17	0.000	[−0.23, −0.11]	−0.09	0.007	[−0.15, −0.03]
*β_1_ + β_3_*	−0.15	0.000	[−0.20, −0.11]	−0.15	0.000	[−0.20, −0.09]
The average LOS of inpatient care (day)						
*β_0_*	9.89	0.000	[9.73, 10.05]	8.57	0.000	[8.10, 9.04]
*β_1_*	−0.02	0.000	[−0.03, −0.01]	0.00	0.845	[−0.03, 0.03]
*β_2_*	0.09	0.356	[−0.11, 0.30]	0.44	0.211	[−0.26, 1.14]
*β_3_*	−0.05	0.000	[−0.07, −0.02]	0.04	0.337	[−0.04, 0.12]
*β_1_ + β_3_*	−0.07	0.002	[−0.09, −0.05]	0.04	0.269	[−0.03, 0.12]

*β_0_*, the baseline level; *β_1_*, the baseline trend prior to intervention; *β_2_*, the immediate level of change after the intervention compared to the pre-intervention; *β_3_*, the trend change after the intervention compared to the pre-intervention; *β_1_*+ *β_3_*, the trend after the intervention.

### Changes to the proportion of the OOP expenditure in inpatient medical costs

**Figure 3:**
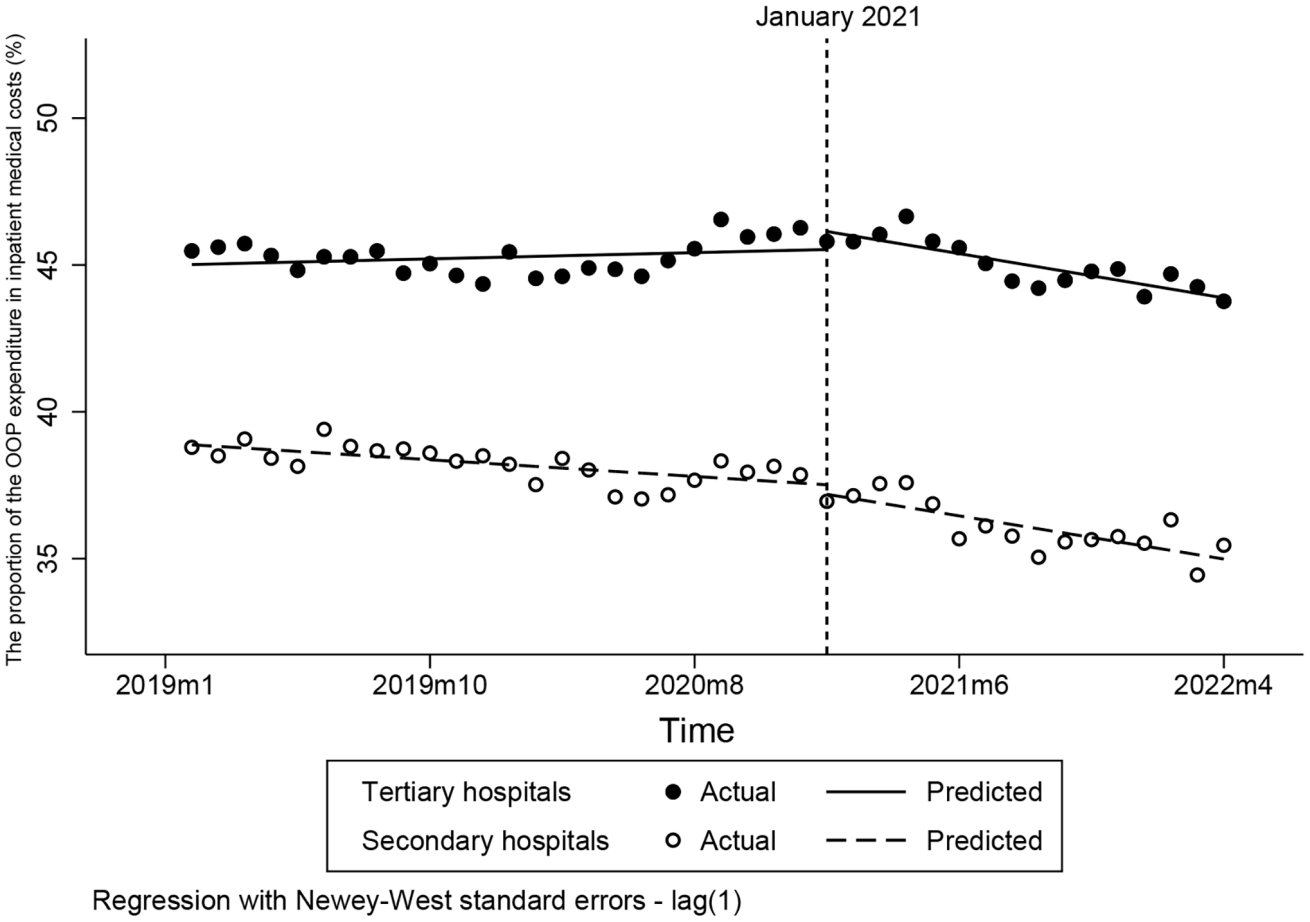
Changes to the proportion of the OOP expenditure in inpatient medical costs. OOP, out-of-pocket.

After the intervention, the proportion of the OOP expenditure in inpatient medical costs in secondary and tertiary hospitals both decreased slightly (*p* < 0.001) (Figure 3). The proportion of the OOP expenditure in inpatient medical costs in secondary decreasing by 0.09 percentage points per month compared to the pre-intervention trend (*p* < 0.01). Furthermore, the reduction in the proportion of the OOP expenditure in inpatient medical costs in tertiary hospital was more pronounced than that in secondary hospital (Table 3), decreasing by 0.17 percentage points per month compared to the pre-intervention trend (*p* < 0.001) (Table 3).

### Changes to the average LOS of inpatient care

**Figure 4:**
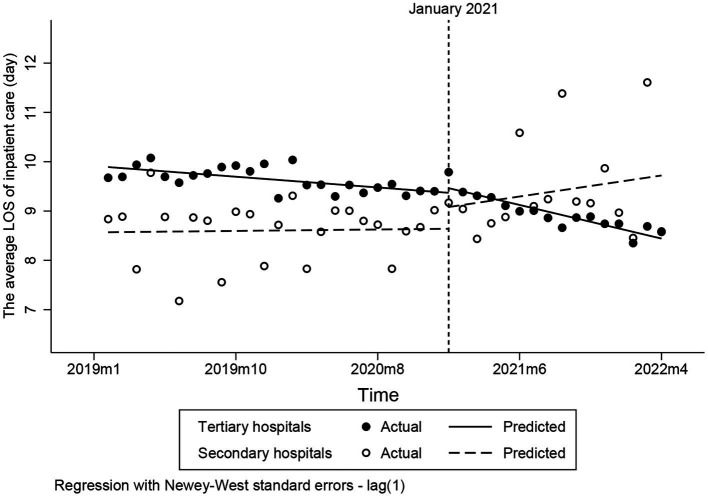
Changes to the average LOS of inpatient care. LOS, length of stay.

Prior to intervention, the baseline trend of the average LOS of inpatient care in secondary and tertiary hospitals had no obvious difference (Figure 4). After the intervention, the average LOS of inpatient care in secondary immediately increase 0.44 day in February 2021 which is significantly obvious than that in tertiary hospitals (*p* = 0.211). However, the change in average LOS of inpatient care in secondary and tertiary hospitals were opposite. Compared to the pre-intervention trend, the average LOS of inpatient care in tertiary hospital decreased by 0.05 day (*p* < 0.001), and the average LOS of inpatient care in secondary hospital increased 0.04 day (*p* < 0.001) (Table 3).

## Discussion

The rapid growth of the inpatient health expenditure poses a serious challenge for the Chinese government (25). Faced with this predicament, the Chinese government has implemented a series of healthcare provider payment reform policies including the DIP payment reform, especially since the National Healthcare Security Administration was established in 2018 (26).

In this study, we found that the inpatient medical costs per case decreased after the implementation of the DIP payment reform, which was aligned to the related studies (17). Consequently, this result is believed to illustrate the advantage of the DIP payment reform design in controlling the inpatient medical costs, especially for tertiary hospitals. Based on the design of the DIP payment, in the situation where the unit price of each score in the DIP payment was unknown before the end of a year, hospitals under the DIP payment had to improve the efficiency of their inpatient care in order to prevent the potential money devaluation of scores of DIP disease groups. Furthermore, although the detailed classifications of DIP disease groups make the provider payment more rational and scientific, however it also brings higher requirement to capacity of cost containment and management of different hospitals. In this case, the hospitals especially tertiary hospitals under the DIP payment will have more initiatives to control the medical costs to ensure the normal revenue of hospitals.

Previous studies highlight that the single healthcare provider payment reforms occasionally result in the increase in the proportion of the OOP expenditure or other medical costs of healthcare services (27, 28). In China, the healthcare service purchasers, such as the Healthcare Security Administration, preferred to use the single healthcare provider payments, such as the global budget or quota payments to pay for hospitals through “premium packages” (7, 29). Additionally, the payment standards of single healthcare provider payments used in China commonly lack the detailed risk-adjustment mechanisms that generate payment standards, which cannot satisfy the practical demand of different hospitals (30). Therefore, hospitals have had to decrease the provision of healthcare services reimbursed by medical insurance to prevent overspending of the medical insurance budget. In China, due to the service volume in tertiary hospitals is much larger than that in secondary hospitals, therefore that phenomenon is more obvious in tertiary hospitals (31). Surprisingly, for both secondary and tertiary hospitals, the proportion of the OOP expenditure in inpatient medical costs was found to significantly decrease after the intervention, fully illustrating that the implementation of the DIP payment reform could effectively promote hospitals to increase the provision of healthcare services covered by medical insurance and regulate the provider behavior in hospitals. It is concluded that the average score of the disease groups, along with the risk-adjustment mechanism of payment standards, are the key factors contributing to this effect. The establishment of the risk-adjustment mechanism aligns the payment standards more closely with the practical demand of secondary and tertiary hospitals, thereby alleviating the pressure of cost containment while providing more rational treatments and services to patients. In addition, this result convincingly proves that the risk-adjustment factors mechanism can promote payment standards more reasonable, which can better satisfy the actual operation costs of hospitals with different statuses.

It is worth noting that the average LOS in secondary hospital experienced an obvious increase after the DIP payment reform was implemented, which was opposite to that in tertiary hospital. We consider that this opposite result is a positive reaction to the implementation of the DIP payment. In order to better reflect the service capacity of hospitals, the reimbursements of hospitals in the DIP payment are relevant to the severity degree of inpatient cases and the status of hospitals. It not only promoted hospitals have more initiatives to treatment the sicker cases, but also achieved that the hospitals under the DIP payment have no necessary to strictly control the LOS of inpatient care due to the cost containment. Accordingly, the reasons we conclude behind the change of the average LOS in this study was that the implementation of the DIP payment enhanced the enthusiasm of inpatient care provision in secondary hospitals, which made some patients who can be treated in secondary hospitals accept more treatments. Additionally, the number of secondary hospitals is more than that of tertiary hospitals, and the copayment ratio of inpatient care in City A is increased with the status of hospitals increases. In this case, the patients got more inpatient care in secondary hospitals also led to the LOS in tertiary hospitals decreasing. Furthermore, the reduction of the proportion of the OOP expenditure in inpatient medical costs in tertiary hospitals compared to the pre-intervention trend is more obvious than that in secondary hospitals after the DIP payment reform was implemented, which further illustrates that the DIP payment reform can effectively alleviate the cost-control pressure of inpatient care in tertiary hospitals. Therefore, combined with the change in the proportion of OOP expenditure in inpatient medical costs and average LOS in secondary and tertiary hospitals, we believe that the DIP payment reform not only alleviated the provision pressure of inpatient care in tertiary hospitals but also improved the rational allocation of the regional healthcare resources. However, Vuagnat et al. outlined that case-based payments, such as the DRG payment, could indirectly lead hospitals to increase the average LOS and readmission rates of inpatient care for the sake of getting more premium (32). Furthermore, related study highlighted that the implementation of the DIP payment could potentially increase the frequency of up-coding behavior in hospitals (17). According to the change in average LOS in this study, we cannot rule out the possibility that the implementation of the DIP payment reform might also generate unintended consequences for inpatient care in secondary hospitals.

## Limitations

This study has several limitations that needed to be addressed: First, this study did not introduce a control group which means that we cannot rule out the possibility that the estimated changes are due to the impact of other phenomena, that changed patient and hospital behavior. Second, due to its recent introduction, we were only able to assess the short-term effects of the DIP payment reform, yet it is possible that there may be a lag in realizing the full effect of policy changes as patients and providers adapt to the new incentives. Third, this study did not involve patient outcomes, such as patient satisfaction or readmission rate, which could indicate a potential impact of the DIP payment on quality of inpatient care.

## Conclusion

The DIP payment could effectively regulate provider behavior of inpatient care in hospitals in the short term, both of which are clearly reflected in the decrease of inpatient medical costs per case and the proportion of the OOP expenditure in inpatient medical costs in secondary and tertiary hospitals. Meanwhile, the implementation of the DIP payment could improve the rational allocation of the regional healthcare resources, which is reflected in the significant increase of the average LOS of inpatient care in secondary hospital and the decrease of average LOS of inpatient care in tertiary hospital. However, the long-term effects of the DIP payment reform still need to be evaluated in the future. In summary, we suggest the countries that are using the case-based payment or ongoing provider payment reform of inpatient care refer to the design elements of the DIP payment to further improve the design of case-based payments.

## Data availability statement

The data analyzed in this study is subject to the following licenses/restrictions: the data that support the findings of this study are available from the Healthcare Security Administrations but restrictions apply to the availability of these data, which were used under license for the current study, and so are not publicly available. Requests to access these datasets should be directed to YD, 201920724@mail.sdu.edu.cn.

## Author contributions

YD, CZ, and QS: conceptualization. JY, SD, and QS: project administration and supervision. YD and CZ: data curation, methodology, and software. YD: writing—original draft. YD, SD, and QS: writing—review and editing. All authors contributed to the article and approved the submitted version.

## Funding

This work was undertaken as part of the UK China Health and Economy Partnership, a higher education programme funded by the GlaxoSmithKline (GSK) and supported by the British Council through an unrestricted grant. The funder was not involved in the study design, collection, analysis, interpretation of data, the writing of this article or the decision to submit it for publication.

## Conflict of interest

The authors declare that the research was conducted in the absence of any commercial or financial relationships that could be construed as a potential conflict of interest.

## Publisher’s note

All claims expressed in this article are solely those of the authors and do not necessarily represent those of their affiliated organizations, or those of the publisher, the editors and the reviewers. Any product that may be evaluated in this article, or claim that may be made by its manufacturer, is not guaranteed or endorsed by the publisher.

## Abbreviations

DIP: diagnosis-intervention packet
OOP: out-of-pocket
DRG: diagnosis-related group
ITS: interrupted time series analysis
OLS: ordinary least squares
LOS: length of stay
